# A mechanism for epithelial–mesenchymal transition and anoikis resistance in breast cancer triggered by zinc channel ZIP6 and STAT3 (signal transducer and activator of transcription 3)

**DOI:** 10.1042/BJ20130483

**Published:** 2013-09-27

**Authors:** Christer Hogstrand, Peter Kille, Margaret Leigh Ackland, Stephen Hiscox, Kathryn M. Taylor

**Affiliations:** *Nutritional Sciences Division, King's College London, 3.85 Franklin-Wilkins Building, 150 Stamford Street, London SE1 9NH, U.K.; †Department of Biosciences, Cardiff University, Main Building, Museum Avenue, Cardiff, CF10 3AT, U.K.; ‡School of Life and Environmental Sciences, Burwood Campus, Deakin University, 221 Burwood Highway, Burwood, VIC 3125, Australia; §Breast Cancer Molecular Pharmacology Unit, School of Pharmacy and Pharmaceutical Sciences, Redwood Building, Cardiff University, King Edward VIIth Avenue, Cardiff CF10 3NB, U.K.

**Keywords:** breast cancer, cell detachment, epithelial–mesenchymal transition (EMT), LIV-1, signal transducer and activator of transcription 3 (STAT3), solute carrier family 39, member 6 (SLC39A6), ZIP6, CHO, Chinese-hamster ovary, *C*_T_, threshold cycle value, E-cadherin, epithelial cadherin, EGF, epidermal growth factor, ELP2, elongation protein 2, EMT, epithelial–mesenchymal transition, ER, oestrogen receptor, FAS, fulvestrant, GSK, glycogen synthase kinase, qPCR, quantitative real-time PCR, SC, cytoplasmic loop between TM3 and TM4, SLC39, solute carrier family 39, STAT3, signal transducer and activator of transcription 3, STATIP1, STAT3-interacting protein 1, TAM, 4-hydroxytamoxifen, TGF, transforming growth factor, TM, transmembrane, β-TrCP, β-transducing repeat-containing protein, ZIP, Zrt- and Irt-like proteins

## Abstract

Genes involved in normal developmental processes attract attention as mediators of tumour progression as they facilitate migration of tumour cells. EMT (epithelial–mesenchymal transition), an essential part of embryonic development, tissue remodelling and wound repair, is crucial for tumour metastasis. Previously, zinc transporter ZIP6 [SLC39A6; solute carrier family 39 (zinc transporter), member 6; also known as LIV-1) was linked to EMT in zebrafish gastrulation through a STAT3 (signal transducer and activator of transcription 3) mechanism, resulting in nuclear localization of transcription factor Snail. In the present study, we show that zinc transporter ZIP6 is transcriptionally induced by STAT3 and unprecedented among zinc transporters, and is activated by N-terminal cleavage which triggers ZIP6 plasma membrane location and zinc influx. This zinc influx inactivates GSK-3β (glycogen synthase kinase 3β), either indirectly or directly via Akt or GSK-3β respectively, resulting in activation of Snail, which remains in the nucleus and acts as a transcriptional repressor of E-cadherin (epithelial cadherin), CDH1, causing cell rounding and detachment. This was mirrored by ZIP6-transfected cells which underwent EMT, detached from monolayers and exhibited resistance to anoikis by their ability to continue proliferating even after detachment. Our results indicate a causative role for ZIP6 in cell motility and migration, providing ZIP6 as a new target for prediction of clinical cancer spread and also suggesting a ZIP6-dependent mechanism of tumour metastasis.

## Abstract 2

We demonstrate a novel mechanism for the ability of cellular zinc to drive cell detachment and migration with implications for breast cancer spread. This mechanism involves a zinc uptake channel ZIP6 (also known as SLC39A6) and a transcription factor, STAT3.

## INTRODUCTION

Genes involved in normal developmental processes are attracting attention as mediators of tumour progression as they facilitate migration and invasion of epithelial tumour cells. EMT (epithelial–mesenchymal transition), an essential part of embryonic development, tissue remodelling and wound repair, is crucial for tumour metastasis [1]. During EMT, cellular adhesion is lost, the cytoskeleton is remodelled and mesenchymal indicative genes are expressed allowing cells to migrate and detach.

Zinc is essential for life with zinc deficiency causing stunted growth and serious metabolic disorders [2]. Apart from the traditional longer-term transcriptional effects of zinc, previous evidence shows zinc can behave as a second messenger [3], manifested by zinc release from intracellular stores within minutes of an extracellular stimulus [4], extending its role in cell signalling to that comparable with calcium. Zinc channel ZIP6 [SLC39A6; solute carrier family 39 (zinc transporter), member 6; also called LIV-1] is a member of the ZIP (Zrt- and Irt-like proteins; now termed SLC39A) family of zinc transporters that influx zinc into the cell [5] and play a major role in intracellular zinc homoeostasis. The SLC39A family of zinc transporters are now considered channels as the prokaryotic homologue of this family, ZIPB, translocates zinc in a non-saturable fashion, indicating that ZIP transporters can function as Zn^2+^ ion channels by transporting zinc down a concentration gradient [6]. Furthermore, the exact mechanism of ZIP7 channel gate opening has been shown recently to be regulated by phosphorylation by protein kinase CK2 [4]. This family of one subfamily of the SLC39A zinc transporters, termed the LIV-1 subfamily and defined by a highly conserved novel potential metalloprotease motif [7], contains nine human members, many of which are increasingly being implicated in a variety of disease states [8]. ZIP6, the first LIV-1 family member to be described, is associated with ER (oestrogen receptor)-positive breast cancer [9], lymph node spread [10] and is a marker of ER-positive (Luminal A) cancers [11,12]. The Oncomine database of clinical samples significantly places ZIP6 in the top 10% of genes overexpressed in ER- and lymph-node-positive breast cancers, a relationship verified in 14 separate clinical studies. Importantly, using anti-ZIP6 antibodies, we have confirmed this in cell lines and clinical material [8].

ZIP6 is known to be involved in EMT and cell migration during early embryonic development [13] in a mechanism involving the transcription factors STAT3 (signal transducer and activator of transcription 3) and Snail. During gastrulation in zebrafish, STAT3 transactivates expression of Zip6, which promotes nuclear localization of Snail and results in repression of *cdh1* [E-cadherin (epithelial cadherin)] expression and increased cell migration [13]. Both STAT3 and Snail have proven roles in cancer metastasis [14]. Furthermore, in a small series of clinical breast cancers [8], ZIP6 was associated with prognostic indicators of cancer development and demonstrated a significant correlation between STAT3 and ZIP6 expression (*P*<0.007), suggesting a mechanistic link between zebrafish gastrulation and breast cancer spread. Further evidence for an involvement of ZIP6 with Snail was provided by the observation that siRNA for ZIP6 reduced HeLa cell invasion via a Snail pathway [15]. Taken together, these findings suggest that ZIP6 could form a link between cancer and normal development [16], where metastatic breast cancer cells reactivate the ZIP6-dependent EMT programme essential for the early embryo.

STAT3 has also been linked to EMT [17], is constitutively activated in many human malignancies, has a well-defined link to breast cancer progression [18] and is required for survival of cultured tumour cells [14,19,20]. Tyrosine phosphorylation of STAT3 on Tyr^705^ triggers its dimerization and translocation to the nucleus where it activates genes involved in cell proliferation, apoptosis, angiogenesis, cell invasion, metastasis and immune function [21].

Transcription factor Snail plays a significant role in EMT by preventing expression of epithelial character genes such as E-cadherin and encouraging transcription of mesenchymal character genes such as vimentin [22], causing loss of adherence and enabling movement to a new site, mirroring metastasis in cancer. GSK-3β (glycogen synthase kinase 3β) deactivates Snail by phosphorylation, promoting nuclear export and priming it for ubiquitination by β-TrCP (β-transducing repeat-containing protein; also known as BTRC] and subsequent degradation [23]. Zinc transporter ZIP6 is essential for the nuclear localization of Snail [13], suggesting that it plays an important role in EMT which may be manifested by ZIP6-mediated zinc influx and the subsequent zinc-mediated inhibition of GSK-3β [24,25].

In the present study, we show that, unprecedented among other zinc transporters, ZIP6 is expressed as a pro-protein and is N-terminally cleaved before relocation to the plasma membrane. Additionally, cells expressing plasma-membrane-located ZIP6 undergo EMT and detach from monolayers as highly proliferating cells. As in the zebrafish embryo, this process is driven by STAT3 and involves activation of Snail, leading to loss of cell adherence by Snail-mediated down-regulation of E-cadherin. We provide evidence for a mechanism where ZIP6-induced zinc influx inactivates GSK-3β, enabling unphosphorylated Snail in the nucleus to down-regulate adherence genes such as E-cadherin, causing loss of cell adherence. This mechanism explains the loss of cell adherence in ZIP6-positive cells and provides a major advance in our understanding of both normal cell processes, such as embryonic development and also disease states such as cancer.

## MATERIALS AND METHODS

### Materials and antibodies

In-house anti-ZIP6 antibodies were generated: anti-rabbit polyclonal antibody against peptide HHDHDHHSDHEHHSD (residues 93–107 on the N-terminus) termed ZIP6-M, and anti-mouse monoclonal antibody against peptide VSEPRKGFMYSRNTNEN (residues 238–254 on the N-terminus) termed ZIP6-Y (see Figure 3D). Rabbit anti-ZIP6-SC (E-20) antibody against residues 500–550 on the cytoplasmic loop between TM (transmembrane) 3 and TM4, rabbit anti-(STAT3 Ser^727^) (SC-8001-R), rabbit anti-(STAT3 Tyr^705^) (SC-7993-R), rabbit anti-(activated GSK-3α/β Tyr^279^/Tyr^216^) (SC-11758-R) and rabbit anti-E-cadherin (H-108) antibodies were from Santa Cruz Biotechnology, and rabbit anti-Snail antibody was from Abgent. Rabbit anti-STATIP1 (STAT3-interacting protein 1) (ab154643) antibody was from Abcam, mouse anti-V5 antibody was from Invitrogen and rabbit anti-V5 antibody was from Bethyl Laboratories. Treatments used were 200 μM STAT3 inhibitor cell-permeable peptide (573096) from Calbiochem, 1 nM oestrogen (E2; oestradiol) from Sigma–Aldrich and 1 μM gefitinib (Iressa, ZD1839) was a gift from AstraZeneca Pharmaceuticals, 10 ng/ml EGF (epidermal growth factor), TGF (transforming growth factor), 100 nM TAM (4-hydroxytamoxifen) and 100 nM FAS (fulvestrant).

### Total RNA extraction from PMC42-LA cells

Total RNA was extracted from PMC42-LA cells [26] using the QIAGEN RNeasy Mini Kit according to the manufacturer's instructions, followed by DNA removal using the Ambion DNA-free™ kit according to the manufacturer's instructions. RNA concentrations and quality were estimated using a DU 530 Life Science UV spectrophotometer (Beckman) at 260 nm/280 nm wavelengths. Total RNA was standardized to 5000 ng/ml for cDNA synthesis.

### qPCR (quantitative real-time PCR) amplification

Amplification reactions were performed with 1×SYBR Green PCR Master Mix (Applied Biosystem), 3 mM of forward and reverse primers and 200 ng of cDNA (for primer sequences see Supplementary Table S1 at http://www.biochemj.org/bj/455/bj4550229add.htm). Samples were analysed in triplicate using GeneAmp 5700 Sequence Detection System (PE Biosystems). The *C*_T_ (threshold cycle value), the cycle number when fluorescence level exceeds the threshold value, was calculated after each reaction. The *C*_T_ of GAPDH (glyceraldehyde-3-phosphate dehydrogenase) was subtracted from the *C*_T_ of the target gene to produce Δ*C*_T_ for each sample. The relative RNA expression level of each sample was calculated using the equation 2^−ΔΔ*C*_T_^, where ΔΔ*C*_T_ is the difference between the treated Δ*C*_T_ and control Δ*C*_T_.

### Cell lines and immunocytochemistry

MCF-7 [27] and CHO (Chinese-hamster ovary) [28] cells were cultured as described previously. For immunohistochemistry, cells were seeded on to sterile 3-aminopropyl-triethoxy-silane-coated glass coverslips, allowed to grow to log phase and fixed using 3.7% formaldehyde with a post-fixation step in methanol/acetone at −20°C, before immunostaining and detection of bound antibody by HRP (horseradish peroxidase)-conjugated avidin–biotin antibody system in conjunction with a DAB (diaminobenzidine) tetrahydrochloride substrate.

### Transfections and invasion assays

The generation of recombinant constructs for ZIP6/LIV-1/SLC39A6 [29], ZIP7/HKE4 (human KE4) [30] and ZIP14 [28] with C-terminal V5-tags using vector pcDNA3.1/V5–His-TOPO (Invitrogen) has been described previously. Cells were transfected with Lipofectamine™ 2000 (Life Technologies) for 16 h with the addition of 3 mM sodium butyrate to cells transfected with ZIP6 and ZIP10 for 14 h before harvest. For transient siRNA transfections, SMARTpool siRNA (100 nM) for ZIP6 and STAT3 (Dharmacon) was used with siRNAMAX lipid transfection reagent (Invitrogen) according to the manufacturer's instructions and harvested after 72 h. Invasion assays of MCF-7 cells across Matrigel™ have been described previously [31]. In this case, transiently transfected cells were transferred to invasion chambers 8 h post-transfection and harvested 16 h later.

### SDS/PAGE and Western blot analysis

Cells were harvested, washed with PBS, lysed for 1 h at 4°C with 5.5 mM EDTA, 0.6% Nonidet P40 and 10% mammalian protease inhibitor cocktail (Sigma–Aldrich) in Krebs–Ringer Hepes buffer [30] and centrifuged at 15000 ***g*** for 15 min at 4°C. Protein was measured using the DC assay kit (Bio-Rad Laboratories) and 20 μg was run per lane under reducing conditions. Transfected CHO cells were harvested between 16 and 24 h after transfection as described previously [29]. Quantification of Western blot results was performed by normalization of three separate experiments to β-actin values.

### Fluorescent microscopy and FACS analysis

Cells (2.2×10^5^) were grown on 0.17-mm-thick coverslips for 24 h before transfection. Coverslips were fixed and processed as described previously [29]. For FACS analysis, adherent cells, harvested carefully by pipette, or non-adherent cells were resuspended in Krebs–Ringer Hepes buffer, incubated with antibodies for 1 h, followed by Alexa Fluor® 488-conjugated secondary antibody for 30 min on ice. A BD Biosciences FACS III flow cytometer and software was used to analyse the FACS results. Cell-cycle analysis was performed using Cycletest™ Plus DNA reagent kit (BD Biosciences) following the manufacturer's instructions.

### Prediction of STAT3- and ER-binding motifs in the 5′ regulatory regions of *ZIP6* and *ELP2*

The 20.9 kb region between the translation start sites of human *ZIP6* and *ELP2* (Chromosome 18: 33706970–33709897) was retrieved from Ensembl (Genome assembly: GRCh37). The sequence was analysed using the online TFBIND software (http://tfbind.hgc.jp/), which uses weight matrix in transcription factor database TRANSFAC R.3.4. The cut-off was 0.71 for STAT3 (Matrix: M00225) and 0.73 for ER (Matrix: M00191). Conservation of transcription factor binding sites was investigated using the ECR (evolutionary conserved regions) Browser in dCode (http://www.dcode.org/).

### Statistical analysis

Statistical analysis was performed using ANOVA with post-hoc Dunnett test. Significance was assumed with *P<0.05, **P<0.01 and ***P<0.001. Error bars show S.D. for at least three different experiments.

## RESULTS

### Relationship between ZIP6 and STAT3 expression

PMC42-LA breast cells can progress on to EMT on treatment with EGF for 3 days [26]. To investigate the effect of EMT stimulation on ZIP6, qPCR analysis of ZIP6, STAT3, Snail and E-cadherin transcripts were measured after 3 days of treatment with 10 ng/ml EGF. The results shown in Table 1 demonstrate significant increases in mRNA (**P<0.001) of ZIP6 (25-fold), STAT3 (6-fold) and Snail (5-fold) with a corresponding decrease in E-cadherin (7-fold) on stimulation of EMT.

**Table 1:** Increased ZIP6 RNA expression in PMC42-LA breast cells in EMT

RNA expression	Control	EGF**
STAT3	1±0.1	6.5±0.1
ZIP6	1±0.25	25.1±1.4
Snail	1±0.1	4.8±0.5
E-Cadherin	1±0.01	0.14±0.1

**P<0.001.

**Figure 1:**
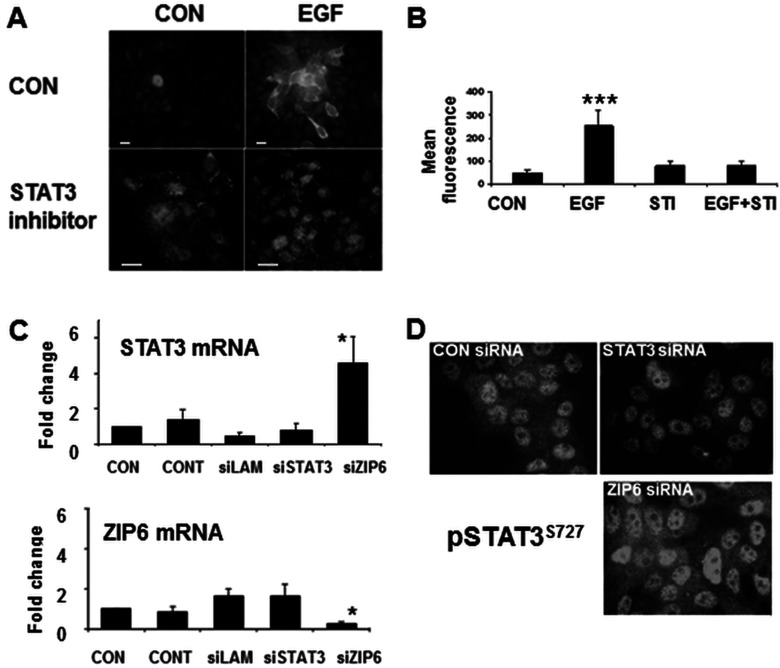
ZIP6 expression requires STAT3 Fluorescent microscopy (**A**) or FACS analysis (**B**) of MCF-7 cells treated with EGF and/or a STAT3 inhibitor (STI) for 24 h and probed with ZIP6-Y antibody. The observed increase in ZIP6 by EGF treatment is prevented by the STAT3 inhibitor. FACS results show mean fluorescence±S.D. for three experiments. Statistical significance of EGF compared with control of *P*<0.001 is indicated by asterisks (***). (**C**) siRNA transfection of MCF-7 cells with lipid control (CON), Lamin siRNA (LAM), STAT3 siRNA or ZIP6 siRNA for 72 h were tested for STAT3 and ZIP6 expression. Results are means±S.D. for six experiments expressed as percentage control. siRNA for ZIP6 increased the expression of STAT3. (**D**) siRNA transfected MCF-7 cells on coverslips for 72 h were fixed and stained for STAT3 pSer^727^ (pSTAT3^S727^) conjugated with Alexa Fluor® 594. pSTAT3^S727^ was reduced when treated with siRNA for STAT3 and increased when treated with siRNA for ZIP6.

Stimulation of ZIP6 expression in MCF-7 cells by EGF treatment for 24 h is inhibited by a STAT3 inhibitor (Figure 1A), suggesting a STAT3-dependent mechanism for ZIP6 expression, and confirmed by parallel FACS analysis (Figure 1B).

In order to investigate further the relationship between ZIP6 and STAT3 gene expression, we used siRNA to ZIP6 or STAT3 to knock down expression of the respective genes and tested for effects on expression of the other gene. These results are the average of six experiments expressed as percentage control±S.D. We observed a significant reduction in ZIP6 gene expression in the presence of ZIP6 siRNA demonstrating the efficacy of the treatment, but no alteration in ZIP6 expression in the presence of siRNA for STAT3, suggesting that constitutive expression of ZIP6 does not require STAT3 (Figure 1C). However, we did observe a significant increase in STAT3 mRNA in the presence of ZIP6 siRNA, suggesting that ZIP6 may have a role in inhibiting STAT3 mRNA expression, which was also evident at the protein level (Figure 1D). Cells treated with siRNA for ZIP6 for 72 h show increased intensity of staining for phosphorylated STAT3.

### Association of ZIP6 with ELP2 (elongation protein 2) and E-cadherin

**Figure 2:**
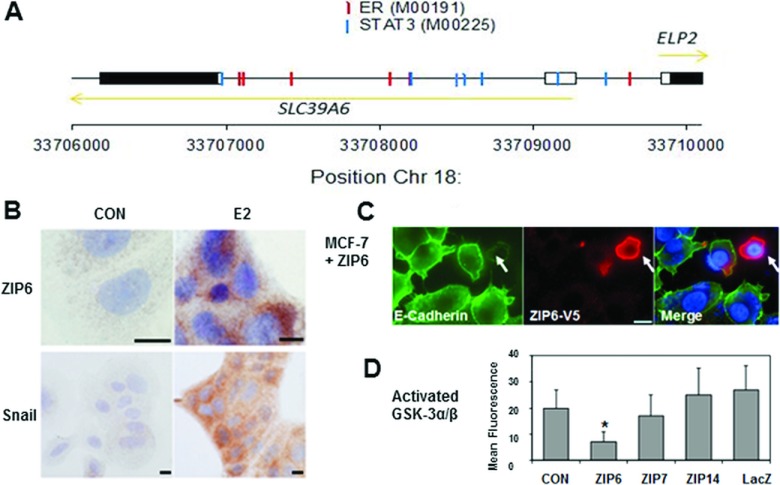
*ZIP6* and *ELP2* are regulated by a shared 5′ region (**A**) Annotated genomic region on human chromosome 18, including the 5′-teminal regions of *SLC39A6* (*ZIP6*) and *ELP2* (*STATIP1*). Coding regions are shown as filled boxes and UTRs of exons as open boxes. Motifs for ER (Transfac ER motif M00191) and STAT3 (Transfac STAT3 motif M00225) are denoted by red and blue vertical lines respectively. The direction of the two genes in the genomic sequence is shown by golden arrows. (**B**) Immunocytochemical analysis showing increase in ZIP6 and Snail in MCF-7 cells treated with oestrogen (E2) for 7 days (brown). The nucleus was counterstained blue. (**C**) Loss of E-cadherin (green) only in recombinant ZIP6-positive cell probed with anti-V5 antibody (red). (**D**) MCF-7 transfected cells were probed for activated GSK-3β and analysed by FACS analysis using Alexa Fluor® 488. Results are means±S.D. for three experiments. Only cells transfected with ZIP6 show inhibition of GSK-3β. CON, control.

The position of the start of the ZIP6 gene in chromosome 18 is 682 bp from the start of ELP2 (or STATIP1) which is transcribed in the opposite direction (Figure 2A). A motif search for transcription factor binding sites revealed seven high-scoring (0.71–0.76) STAT3 motifs in addition to six putative binding sites (score: 0.74–0.84) for ER (Figure 2A). One STAT3 site was located within 179 bp of the evolutionarily conserved region including the untranslated exon 1 of *zip6* and was conserved in the mouse and zebrafish genome (Supplementary Figures S1 and S2 at http://www.biochemj.org/bj/455/bj4550229add.htm). The experimentally confirmed response of the *zip6* gene to STAT3 and oestrogen combined with the presence of high-confidence predicted binding sites, suggest that STAT3 and ER directly mediate expression of *zip6.*

We demonstrate a link between ZIP6 and Snail using immunocytochemistry, where they both increase in response to oestrogen (Figure 2B). Furthermore, we show a loss of E-cadherin in a ZIP6-positive cell (arrow) as a direct downstream effect of Snail acting as a nuclear transcription factor (Figure 2C). As GSK-3β deactivates Snail by phosphorylation [23] we tested transfected cells for activated GSK-3α/β by FACS analysis (Figure 2D) and showed a significant reduction in activated GSK-3α/β present in ZIP6-transfected cells compared with controls. These results are consistent with ZIP6-mediated zinc influx causing an inhibition of GSK-3α/β allowing Snail activation and causing E-cadherin loss.

### Zinc transporter ZIP6 is cleaved on the N-terminus before locating to the plasma membrane

**Figure 3:**
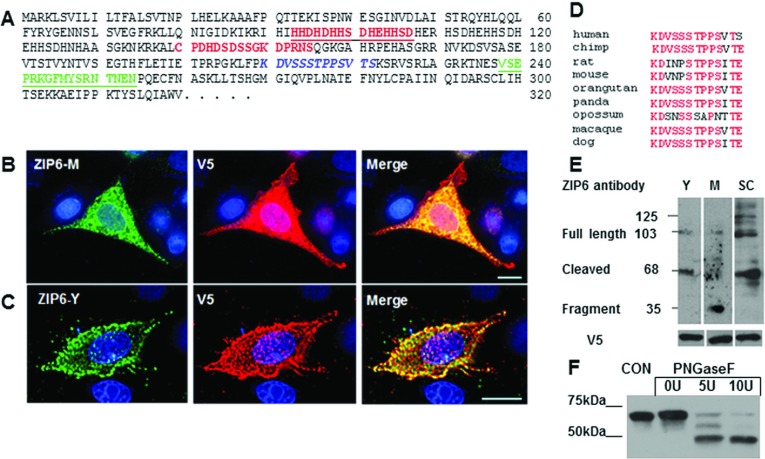
ZIP6 is a pro-protein and N-terminally cleaved before relocating to the plasma membrane (**A**) N-terminal 320 residues of ZIP6 sequence showing position of antibody epitopes. Residues 93–107 is ZIP6-M antibody (red underlined), antibody to residues 140–155 (red) stains endoplasmic reticulum ZIP6 [41], ZIP6-Y antibody against residues 238–254 and the mouse equivalent stains plasma membrane ZIP6 in mouse brain [49]. Residues 210–222 (blue italic) marks a strongly predicted PEST proteolytic cleavage site [29]. ZIP6 transfected cells were probed with anti-V5 antibody conjugated with Alexa Fluor® 594 and either ZIP6-M (**B**) or ZIP6-Y (**C**) antibodies conjugated with Alexa Fluor® 488. ZIP6-M antibody does not recognize plasma-membrane-located ZIP6 in permeabilized cells (**B**), whereas ZIP6-Y does in non-permeabilized cells (**C**). (**D**) Conservation of potential PEST site in mammalian ZIP6 sequences. (**E**) Different ZIP6 antibodies generate different molecular mass bands. Full-length ZIP6 is 103 kDa, the cleaved N-terminal fragment is 35 kDa and ZIP6 after cleavage is 68 kDa. These samples were run on the same blot which was cut to probe with different antibodies. (**F**) MCF-7 cells treated with 0, 10 or 20 units PNGaseF for 3 h at 37°C and probed with ZIP6 SC antibody show a reduction in size of the 68 kDa band consistent with two glycosylated side chains. Molecular masses are indicated in kDa. CON, control.

Generation of anti-ZIP6 antibodies has highlighted that endoplasmic reticulum-located ZIP6 is a pro-protein. Our monoclonal antibody against a ZIP6 N-terminal epitope (M, residues 93–107, Figure 3A, red underlined) only stains endoplasmic reticulum ZIP6 and not plasma membrane ZIP6, as verified by the anti-V5 antibody (Figure 3B) in permeabilized cells, confirming that this section of ZIP6 is not present on the plasma membrane, possibly owing to proteolytic cleavage. A second antibody confirmed this conclusion (Y, residues 238–254, Figure 3A, green underlined) with an epitope further downstream, staining plasma membrane ZIP6 (Figure 3C) in non-permeabilized cells. We have previously observed a potential PEST site (residues 210–223, Figure 3A, blue italic) and a potential ubiquitin degradation signal (SSSTPPSV residues 213–220) between these two antibody epitopes [29], suggesting the location of N-terminal cleavage. Both PEST and ubiquitin sites could ensure a protein half-life of less than 1 h [32] and, interestingly, these sites are well-conserved in mammalian ZIP6 sequences (Figure 3D), suggesting an evolutionarily conserved function.

N-terminal proteolytic cleavage of ZIP6 would explain the different ZIP6 bands obtained by Western blot using three anti-ZIP6 antibodies (Figure 3E). We obtained bands of 103 kDa and 35 kDa with ZIP6-M antibody (near N-terminus), 103 kDa and 68 kDa with ZIP6-Y antibody (far N-terminus) and SC antibody (cytoplasmic loop between TM3 and TM4), as well as higher-molecular-mass bands with SC. Full-length unglycosylated ZIP6 is predicted to be 85 kDa and produces a 103 kDa band, consistent with glycosylation [29]. When cleaved, this band would be reduced to 68 kDa leaving the N-terminal fragment of 35 kDa. Furthermore, this 68 kDa band is reduced by 15 kDa in two stages by treatment with PNGaseF (Figure 3F), suggesting two glycosylation side chains on this part of the molecule.

### ZIP6 expression changes STAT3 activation

**Figure 4:**
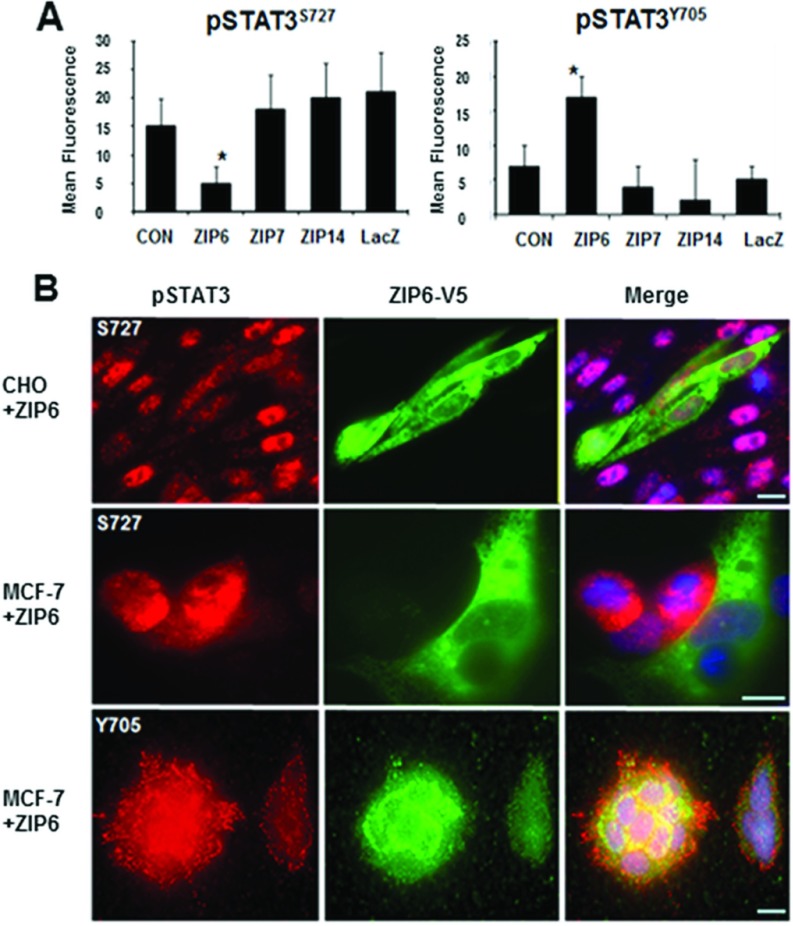
ZIP6 inhibits Ser^727^ and increases Tyr^705^ phosphorylation of STAT3 (**A**) CHO cells transfected with lipid control (CON), ZIP6, ZIP7, ZIP14 and LacZ were probed for either STAT3 pSer^727^ (STAT3^S727^) or STAT3 pTyr^705^ (STAT3^Y705^) conjugated with Alexa Fluor® 488 and analysed by FACS. Cells expressing ZIP6 show a significantly reduced STAT3^S727^ and increased STAT3^Y705^. (**B**) CHO or MCF-7 cells were transfected with ZIP6 visualized with anti-V5 antibody conjugated with Alexa Fluor® 488 (green) and stained with STAT3^S727^ or STAT3^Y705^ conjugated to Alexa Fluor® 594 (red). Nuclei were counterstained with DAPI (blue). ZIP6-positive cells had reduced STAT3^S727^ and increased STAT3^Y705^.

Having linked the ZIP6 and STAT3 gene expression we investigated the relationship between ZIP6 and STAT3 proteins. We examined phosphorylated STAT3 in cells transfected with ZIP6 or other ZIP transporters for comparison. ZIP7 resides on the endoplasmic reticulum and transports zinc out of these stores [4,30], whereas ZIP14 resides on the plasma membrane and transports zinc and other ions into the cell [28]. By FACS analysis we show that STAT3 pSer^727^ was significantly decreased and STAT3 pTyr^705^ significantly increased in cells expressing ZIP6 (Figure 4A) which was further confirmed by fluorescent microscopy (Figure 4B) and in contrast with cells expressing ZIP7, ZIP14 or a LacZ control.

### Recombinant and endogenous ZIP6 is enriched in non-adherent cells

**Figure 5:**
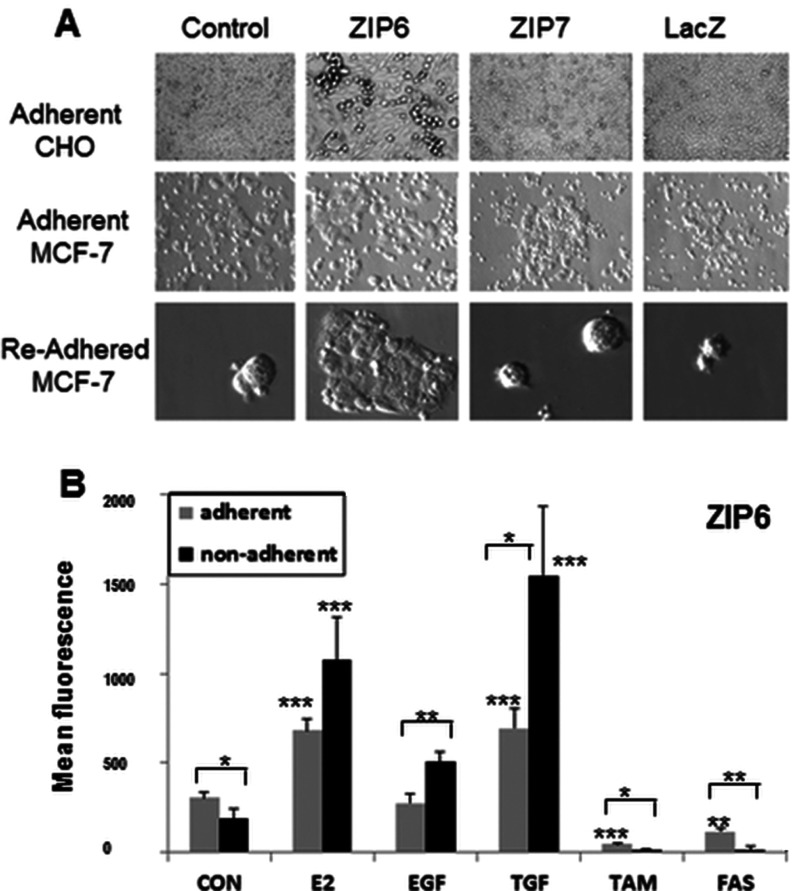
ZIP6 enrichment in non-adherent cells (**A**) CHO or MCF-7 cells transfected with lipid control, ZIP6, ZIP7 or β-galactosidase (LacZ) were imaged 16 h after transfection and cells transfected with ZIP6 had either detached clumps of live cells (CHO) or detached single round cells (MCF-7). Non-adherent MCF-7 cells grown overnight on new coverslips for 24 h (re-adhered) had more cells from ZIP6 transfected dishes. (**B**) Adherent (grey) and non-adherent (black) MCF-7 cells treated for 7 days with oestrogen (E2), EGF, TGF, TAM or FAS (Faslodex®) were collected and probed for ZIP6 conjugated with Alexa Fluor® 488 and analysed by FACS. Results are means±S.D. for three experiments and demonstrate ZIP6 enrichment in non-adherent cells. Statistical significance from the relevant control (CON) is indicated above the histogram bars as *P<0.05, **P<0.01 and ***P<0.001. Comparison between adherent and non-adherent for each treatment is also indicated above this.

We tested whether ZIP6-positive cells were pre-disposed to EMT by collecting the non-adherent cells in culture dishes and applying them to new coverslips and leaving them to attach and grow. Figure 5(A) shows white-light images of cells attached to coverslips, whether 16 h post-transfection (adherent) or non-adherent cells collected at 16 h and re-adhered to coverslips overnight (re-adhered). There are many non-adherent cells present in ZIP6-transfected dishes, either in groups (CHO) or single cells (MCF-7). We confirmed the increased number and viability in ZIP6-transfected non-adherent cells by ability to re-attach on to coverslips.

MCF-7 cells, treated for 24 h with agents to stimulate or inhibit ZIP6 expression, were collected separately as adherent or non-adherent populations and probed for ZIP6 content by FACS analysis. Results demonstrate (Figure 5B) an increase in ZIP6 in adherent cells (grey) treated with oestrogen (E2) and TGF compared with control, and in non-adherent cells (black) treated with E2, EGF and TGF, accompanied by a decrease when treated with anti-oestrogens FAS (Faslodex®) or TAM, consistent with ZIP6 being an E2 regulated gene. Collectively, these results confirm that ZIP6 promotes cell detachment and is enriched in non-adherent cells, consistent with a role of ZIP6 in controlling EMT.

### ZIP6-positive cells detach and have increased motility

**Figure 6:**
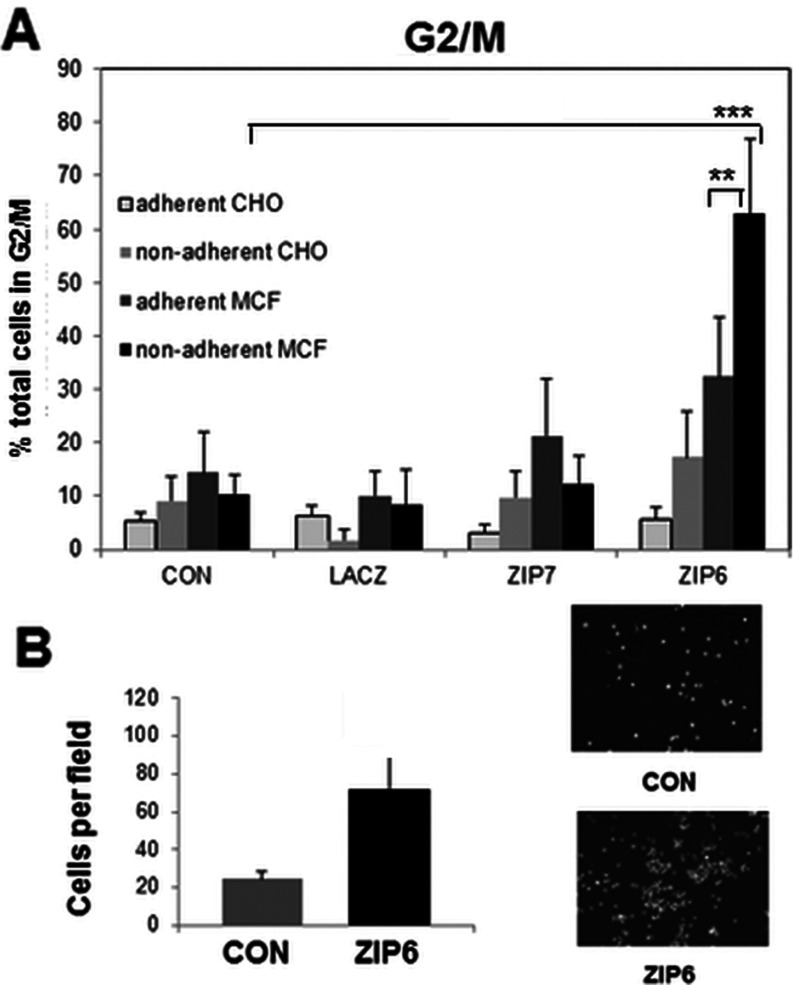
ZIP6-positive cells have increased G_2_/M-phase and increased migration (**A**) CHO and MCF-7 cells transfected with control (CON), LacZ, ZIP7 or ZIP6 were harvested as adherent or non-adherent populations and tested for cell-cycle stage by FACS analysis. Both adherent and non-adherent ZIP6-positive cells had increased cells in G_2_/M-phase suggesting anoikis resistance. Values are means±S.D. for three experiments. Statistical significance of ***P<0.001 was achieved between ZIP6 and control for non-adherent MCF-7 cells and **P<0.005 for ZIP6 adherent and non-adherent cells. (**B**) MCF-7 cells transfected with ZIP6 for 8 h migrated across Matrigel™ on a Transwell plate over the next 16 h more than controls. Images represent migrated DAPI-stained cells. Values are means±S.D. for three experiments.

We further tested the viability of ZIP6-transfected CHO and MCF-7 cells by examining their cell-cycle stage by FACS analysis (Figure 6A). There was little difference in cell-cycle progression in the adherent cell populations, although the ZIP7-transfected MCF-7 cells showed a slight increase in cells in G_2_/M-phase and the ZIP6-transfected cells showed a doubling of cells in G_2_/M-phase, neither was significant. This effect was more marked in the ZIP6-transfected non-adherent cells which were predominantly in G_2_/M-phase. This confirms that ZIP6 is not only enriched in non-adherent cells, but that they are alive and proliferating, suggestive of anoikis resistance.

In order to test the ability of ZIP6 to initiate EMT we investigated whether ZIP6-transfected MCF-7 cells could migrate across Matrigel™ in a transwell plate. Despite the fact that ZIP6 transfection results in less than 10% cells positive for ZIP6 [29], we were able to demonstrate a significant doubling of motility across fibronectin in 16 h in ZIP6-transfected cells (Figure 6B). This result further explains why we previously obtained so few ZIP6-positive cells after 24 h of transfection and obtained relatively more after only 16 h of transfection due to detachment of ZIP6-positive cells.

## DISCUSSION

**Figure 7:**
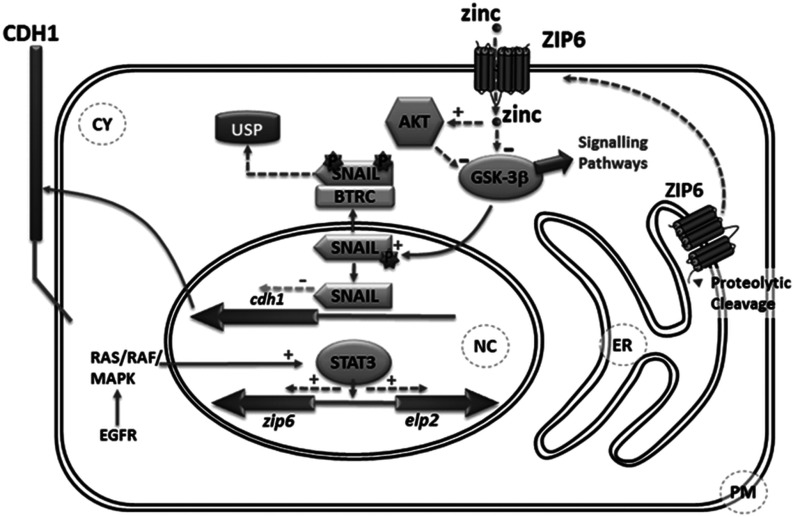
Schematic illustration of proposed ZIP6 signalling pathway Proposed signalling pathway linking zinc channel ZIP6 with loss of adherence through Snail-mediated repression of E-cadherin (CDH1). The N-terminus of ZIP6 located on the endoplasmic reticulum (ER) is proteolytically cleaved triggering translocation to the plasma membrane (PM) facilitating zinc influxes. Zinc entering the cell either activates Akt which inhibits GSK-3β or inhibits GSK-3β directly. Inactive GSK-3β can affect a range of signalling pathways and is unable to phosphorylate and deactivate Snail by β-TrCP-mediated degradation by the ubiquitin-specific peptidase (USP) pathway in the cytosol (CY), increasing the level of nuclear (NC) Snail and down-regulating E-cadherin (CDH1) expression. EGFR, EGF receptor; MAPK, mitogen-activated protein kinase.

Zip6 has been shown previously to be essential for EMT and cell-autonomous movements of gastrula organizer cells in zebrafish [13]. In the present study, we demonstrate that the role of ZIP6 in controlling EMT is conserved between fish and human and unravel details of the Zn^2+^ signalling pathway involved. Human ZIP6 is transcriptionally induced by proliferative factors, such as STAT3 and E2, and appears to be activated through proteolytic N-terminal cleavage before being trafficked to the plasma membrane where it mediates Zn^2+^ influx (Figure 7). This zinc influx triggers phosphorylation of GSK-3β, either directly [25] or indirectly via Akt, which has GSK-3β as a target and is phosphorylated in response to zinc [33]. Inactivation of GSK-3β through phosphorylation renders it unable to phosphorylate Snail, which remains in the nucleus where it acts as a transcriptional repressor of the E-cadherin gene, *CDH1*. Although this is a biological process of critical importance during development [13], it is hijacked by cancer cells to enable them to metastasize and invade tissues. Extracellular zinc can increase cell proliferation and survival [34] through uptake by the zinc-sensing receptor, GPR39 (G-protein-coupled receptor 39), [35] leading to Akt activation. Whether this interacts in any way with ZIP6 on the plasma membrane has yet to be established.

The ability of ZIP6 to promote cell migration of breast cancer cells was coupled with an increased population of non-adherent cells which were enriched for ZIP6, were still viable and actively proliferating. Furthermore, when expression of endogenous ZIP6 was stimulated by E2, EGF or TGFα, the observed increase in ZIP6 was predominantly in the detached cells. Interestingly, transfection of cells with ZIP7 or ZIP14 did not cause cell detachment and this may explain why we previously reported lower transfection efficiency (in adherent cells) for ZIP6 than for ZIP7 and ZIP14 [28,30]. Reducing the time between transfection and harvest to 16 h has increased the number of ZIP6-positive cells obtained by harvesting before detachment. In this context, it is noteworthy that the actions of ZIP6 on tumour cell migration can be mimicked by exposing cells expressing endogenous ZIP6 to exogenous zinc [27]. These data also explain an apparent conflicting result showing parallel levels of ZIP6 and E-cadherin [36]. However, that study transfected cells for 48 h, a time when our data suggest ZIP6-positive cells would have detached. Furthermore, the antibody used was in a similar location to ZIP6-M antibody and our data suggest that it would not recognize plasma-membrane-located ZIP6.

In the present study, we have extended observations by us and others to confirm a relationship between ZIP6 and STAT3 protein suggesting a STAT3/ZIP6-mediated mechanism for cell rounding and detachment that explains the observed presence of ZIP6 in tumours that spread to lymph nodes [10]. This association of zinc transporters with STAT3 is not unprecedented as ZIP14 is regulated by IL-6 (interleukin 6) and STAT3 signalling [37] and ZIP4 activates STAT3 transcription in pancreatic cancer [38]. Furthermore, we observed a positive correlation in a breast cancer series [8] between STAT3 and ZIP6 (*P*=0.007), agreeing with the known association of STAT3 with breast cancer progression [14]. One unexpected result using siRNA to remove either STAT3 or ZIP6 was obtained where ZIP6 removal increased STAT3 expression suggesting ZIP6 modulates STAT3 expression.

We made a novel observation that ZIP6 is modified by N-terminal cleavage before relocation to the plasma membrane which we verified experimentally using our unique ZIP6 antibodies against N-terminal epitopes separated by a potential PEST proteolytic cleavage site [39]. The PEST sequence is commonly associated with proteins of short half-lives [32]. ZIP10, the closest paralogue to ZIP6 and the only other LIV-1 family member with a PEST site, has also been linked to invasive breast cancer [40], but whether ZIP10 is similarly processed is unknown.

This N-terminal cleavage of ZIP6 also explains some apparently conflicting published data. ZIP6 expression levels were associated with good outcome for breast cancer patients, however, the ZIP6 antibody epitope (residues 140–155) would be predicted to recognize endoplasmic-reticulum-located ZIP6 [41]. The mouse ‘ermelin’ protein is equivalent to mouse ZIP6, but is missing the N-terminal 245 residues, the equivalent start site in the human sequence is marked in Figure 3(A) which is notably downstream of the epitope for antibody ZIP6-Y. The remainder of the N-terminal sequence is evident one reading frame away, suggestive of an alternative isoform or a possible sequencing error. However, ermelin was cloned and expressed and found to locate in the endoplasmic reticulum and not the plasma membrane [42], suggesting that N-terminal cleavage is essential for the relocation of ZIP6 to the plasma membrane as it may display an essential signal for trafficking to the plasma membrane. Furthermore, the PEST site found in the N-terminus of ZIP6 is well conserved between species (Figure 3D), suggesting a potential involvement in protein relocation. There are, however, 16 predicted cleavage sites between residues 156 and 237 which are mainly predicted to be cleaved by subtilisin-like pro-protein convertase [43], N-arginine dibasic convertase [44] or general dibasic motifs used as proteolysis signals [45]. The exact site of cleavage is yet to be discovered. To date, few zinc transporters have been demonstrated to be proteolytically modified. These are ZIP4 [46] and ZIP10 [47] and this modification occurred extracellularly.

We demonstrate a reduction in EGF-stimulated ZIP6 protein by a STAT3 inhibitor (Figure 1A). The human *ZIP6* gene, present on the negative strand of chromosome 18, is separated by only 0.8 kb of untranscribed DNA from the gene encoding *ELP2* (alias STATIP1), on the positive strand. As ELP2 can regulate STAT3 as a scaffold protein controlling the ligand-dependent activation of STAT3 [48] and preferentially bind unphosphorylated STAT3 [48], it could provide a mechanism for moderating ZIP6. In fact, the short 5′ sequence between the coding regions of *ZIP6* and *ELP2* has seven putative STAT3-binding sites and one of these, located in the 5′UTR of *ZIP6*, is present in human, mouse and zebrafish, suggesting that STAT3 drives *ZIP6* expression and probably also *ELP2.* Interestingly, we observed increased STAT3 pTyr^705^ and lack of STAT3 pSer^727^ in ZIP6-positive cells, the latter may be indicative of ELP2 binding to unphosphorylated STAT3 [48]. However, the 205 bp exon 1 in part of the 5′UTR is followed by a long (2 kb) intron and translation starts 10 bp into exon 2. As ELP2 inactivates STAT3 by binding to it [48], we speculate that STAT3 may bind to the 682 bp region and drive the expression of both genes.

In summary, we have expanded previous knowledge of the role for the *ZIP6* gene in zebrafish gastrulation, regulation by oestrogen and STAT3 and association with breast cancers that spread to the lymph nodes. In the present study, we have demonstrated that the ZIP6 protein is causative in the ability of human cells to round up, detach and migrate, demonstrating resistance to anoikis, the first step in the process of metastasis, by their ability to remain alive and continue cell-cycle progression. This cell detachment depends on ZIP6 movement to the plasma membrane as a direct result of N-terminal cleavage and subsequent zinc influx which sets in motion a zinc influx/GSK-3β inhibition/Snail activation/E-cadherin loss pathway that results in cell rounding and detachment. These results together suggest that zinc transporter ZIP6 may not only play an important causative role in cell migration, but also be a viable indicator of tumours that have the capability to spread.

## Online data

Supplementary data
