# Supplementary material for: A mechanism for epithelial–mesenchymal transition and anoikis resistance in breast cancer triggered by zinc channel ZIP6 and STAT3 (signal transducer and activator of transcription 3)
Source: Biochem J. 2013 Sep 27;455(Pt 2):229–37. doi: 10.1042/BJ20130483 (PMC3789231; doi:10.1042/BJ20130483)
Supplement: Supplementary data [file bj4550229add.pdf]

BJ Cell

Biochem. J. (2013) **455**, 229–237 (Printed in Great Britain) doi:10.1042/BJ20130483

online data

SUPPLEMENTARY ONLINE DATA

A mechanism for epithelial–mesenchymal transition and anoikis resistance in breast cancer triggered by zinc channel ZIP6 and STAT3 (signal transducer and activator of transcription 3)

Christer HOGSTRAND*, Peter KILLE†, Margaret Leigh ACKLAND‡, Stephen HISCOX§ and Kathryn M. TAYLOR§[1]

*Nutritional Sciences Division, King's College London, 3.85 Franklin-Wilkins Building, 150 Stamford Street, London SE1 9NH, U.K., †Department of Biosciences, Cardiff University, Main Building, Museum Avenue, Cardiff, CF10 3AT, U.K., ‡School of Life and Environmental Sciences, Burwood Campus, Deakin University, 221 Burwood Highway, Burwood, VIC 3125, Australia, and §Breast Cancer Molecular Pharmacology Unit, School of Pharmacy and Pharmaceutical Sciences, Redwood Building, Cardiff University, King Edward VIIth Avenue, Cardiff CF10 3NB, U.K.

> hg19 chr18:33709038-33709216 179bps GC:67.0%
CCCTCCCAGCCGCCCTGCTCCCGGACCTGAAAGACTCACGTCTCCGCGGCCTCGTTGTCCC
ACGGCCCGGCCACGCGCGCAGGTTTGGTTCCACACGGGCGGTCCAGAGGGCTCGGAACG
GGCCCAC**TGGTGTCTTCGAGAAATCTCT**ACCAGGCGCGAACACGCGGTGGTTTCATTGG
> mm10 chr18:24603250-24603421 172bps GC:70.3%
CTGTCCTGCCGCCCCGCTGCGGTCTTGCGGAACTCACCTCTCCTGGTGTCCCGCCGCCCGG
CCCCGCGCGCAGGTTCGGTTCCCCGCGGGAGGTCCGAAGGGACTGGGAACCCGCGCAGC
TCCGGTCTCCGAGAAGCCTTTACGAGGCGTGGAGCAGCGGCAGAATCATGAG

Figure S1 Evolutionary conserved region between human and mouse genomic sequences spanning the 5′UTR of the respective *SLC39A6* gene

A STAT3 site found to be aligned between human and mouse *SLC39A6* is highlighted in bold red font.

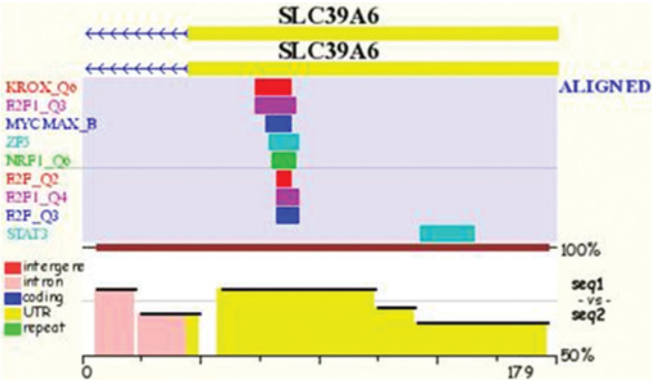

Figure S2 The location of an aligned STAT3 motif is shown as a turquoise horizontal bar

The degree of homology between the two sequences is indicated in the bottom of the Figure on a vertical scale of 50–100%.

[1] To whom correspondence should be addressed (email taylorkm@cardiff.ac.uk).

© 2013 The Author(s)
The author(s) has paid for this article to be freely available under the terms of the Creative Commons Attribution Licence (CC-BY) (http://creativecommons.org/licenses/by/3.0/) which permits unrestricted use, distribution and reproduction in any medium, provided the original work is properly cited.

Biochemical Journal www.biochemj.org

Table S1 Oligonucleotides used for qPCR of PMC42-LA cells

	Forward primer	Reverse primer	PCR product length (bp)
LIV-1	CAGTCACAGCCAGCGCTACTC	ATTGCTAGGCCATCGCTGAA	114
E-Cadherin	TTCCTCCCAATACATCTCCCTT	CGAAGAAACAGCAAGAGCAGCA	76
STAT3	GCCAGAGAGCCAGGAGCA	ACACAGATAAACTTGGTCTTCA	75
Snail1	CAGATGAGGACAGTGGGAAAG	GTAGAGGAGAAGGACGAAGGA	73
GAPDH	CCACCCATGGCAAATTCC	TGGGATTTCCATTGATGACAA	69

Received 5 April 2013/15 July 2013; accepted 7 August 2013
Published as BJ Immediate Publication 7 August 2013, doi:10.1042/BJ20130483

© 2013 The Author(s)
The author(s) has paid for this article to be freely available under the terms of the Creative Commons Attribution Licence (CC-BY) (http://creativecommons.org/licenses/by/3.0/) which permits unrestricted use, distribution and reproduction in any medium, provided the original work is properly cited.
